# Durability of immunity by hepatitis B vaccine in Japanese health care workers depends on primary response titers and durations

**DOI:** 10.1371/journal.pone.0187661

**Published:** 2017-11-09

**Authors:** Nori Yoshioka, Matsuo Deguchi, Hideharu Hagiya, Masanori Kagita, Hiroko Tsukamoto, Miyuki Takao, Hisao Yoshida, Norihisa Yamamoto, Yukihiro Akeda, Yoshiko Nabetani, Ikuhiro Maeda, Yoh Hidaka, Kazunori Tomono

**Affiliations:** 1 Division of Infection Control and Prevention, Osaka University Hospital, Suita, Osaka, Japan; 2 Laboratory for Clinical Investigation, Osaka University Hospital, Suita, Osaka, Japan; 3 Nursing Department, Osaka University Hospital, Suita, Osaka, Japan; University of Cincinnati College of Medicine, UNITED STATES

## Abstract

**Background:**

Health care workers (HCWs) are frequently exposed to hepatitis B virus (HBV) infection. The efficacy and safety of immunization with the hepatitis B (HB) vaccine are well recognized, but the durability of immunity and need for booster doses in those with secondary vaccine response failure remains controversial.

**Methods:**

This was a retrospective cohort study performed at Osaka University Hospital, Japan. We examined antibodies against HB surface antigen (anti-HBs) titers annually after immunization for previously non-immunized HCWs. Primary responders were categorized by their sero-positive durations as short responders (those whose anti-HBs titers declined to negative range within 3 years), and long responders (those who retained positive anti-HBs levels for 3 years and more). We re-immunized short responders with either single or 3-dose boosters, the long responders with a single booster when their titers dropped below protective levels, and examined their sero-protection rates over time thereafter.

**Results:**

From 2001 to 2012, data of 264 HCWs with a median age of 25.3 were collected. The rate of anti-HBs positivity after primary vaccination were 93.0% after three doses (n = 229), 54.5% after two doses (n = 11), and 4.2% after a single dose (n = 24). Of 213 primary responders, the anti-HBs levels of 95 participants (44.6%) fell below the protective levels, including 46 short responders and 49 long responders. HCWs with higher initial anti-HBs titers after primary vaccination had significantly longer durations of sero-positivity. For short responders, 3-dose booster vaccination induced a longer duration of anti-HBs positivity compared to a single-dose booster, whereas for long responders, a single-dose booster alone could induce prolonged anti-HBs positivity.

**Conclusion:**

Our preliminary data suggested that it may be useful to differentiate HB vaccine responders based on their primary response durations to maintain protective levels of anti-HBs efficiently. A randomized, prospective, large-scale study is warranted to support our findings.

## Introduction

Hepatitis B virus (HBV) infection is still a major public health concern worldwide [1]. According to a recent systematic review summarizing publications through 1965 to 2013, the seroprevalence of hepatitis B (HB) surface antigen (HBsAg) was 3.6% worldwide, and approximately 250 million individuals were HBsAg positive globally in 2010 [2]. The virus remains viable for at least a week on environmental surfaces [3] and is transmitted percutaneously, through the mucosa, and even intact skin, as well as via blood and exposure to body fluids. Exposure to HBV is an occupational risk for HCWs; thus, the prevention of HBV transmission in the healthcare setting is a great concern for infection control practitioners in hospitals.

Vaccination is a highly reliable strategy for preventing HBV infection. Universal HB vaccine, which is implemented in over 180 countries as of 2015, has shown promising results in regards to its safety and efficacy [4]. Response rates to primary HB vaccine are high, resulting in approximately 95% of healthy individuals developing protective levels of antibodies to HB surface antigen (anti-HBs) of ≥10 mIU/mL [5]. The vaccination-derived anti-HBs titers, however, wane over time and the need for booster doses for those with anti-HBs negative-conversion has long been debated. It seems plausible that high-risk populations like HCWs may need boosters when their anti-HBs fall below the protective level. Yet, considering the absence of evidence for HBV infection in vaccinated HCWs and the rapid response to a single booster dose [6–8], the Advisory Committee on Immunization Practices (ACIP) [9] and the European Consensus Group on Hepatitis B Immunity [10] do not recommend a routine booster dose for HCWs at present. According to statements by the Center for Disease Control and the World Health Organization (WHO), there is no need to give boosters to individuals who acquired anti-HBs levels of ≥10 mIU/mL after completion of the HB vaccine schedule [8].

We, however, cast doubts on the effectiveness of declined anti-HBs particularly to high-risk populations such as HCWs. Previous reports have described asymptomatic HBV breakthrough infections occurred in persons who had acquired protective levels of anti-HBs after primary vaccination [11, 12]. A recent study with nucleic acid testing revealed that individuals who had been completely vaccinated could still become infected with HBV when anti-HBs titers declined [13]. Additionally, other reports mentioned cases of acute HB [14] and even chronic HB [15] that involved HCWs with dropped anti-HBs levels several years after HB vaccine. Based on this anecdotal evidence, it is questionable whether the vaccine-induced anamnestic immunity is fully protective against HBV infection. The HB vaccine was only integrated into the routine infant immunization program from October 2016 in Japan [16], and thus, recommendations from the authorities may not be followed to the letter.

Our preceding data, though limited by the sample size, suggested that the elevation of anti-HBs titers in those with sero-negative conversion takes place approximately one week after administering a booster. Of the five HCWs whose anti-HBs titers declined after a primary full series of HB vaccine, elevations of anti-HBs over the protective level occurred only 4 days after re-immunization; it took more than 4 days in 2 cases, 7 days in 2 cases, and 8 days in 1 case (**S1 Fig**). According to a previous report that investigated the effectiveness of passive immunity by HB immunoglobulins within 7 days after HBV exposure, clinical hepatitis and anti-HBs seroconversion occurred in 1.4% and 5.6% of exposed HCWs, respectively [17]. Although details were not given, the results of the study indicate that anti-HBs elevation should be achieved no later than one week to prevent HBV infection. Although we need to take the difference in immunogenicity between HB vaccine and HBV infection into consideration, these facts infer that HB vaccine-induced anti-HBs elevation may be inadequate to prevent HBV infection.

This encouraged us to investigate the relevance of initial anti-HBs titers after primary vaccination, to assess the duration of its positivity, and the association between HB vaccine booster administered to those with sero-negative conversion and the duration of anti-HBs positivity thereafter in HCWs. We believe that this study is of great value since most countries have already adopted the HB vaccine as universal immunization, and therefore, it is now difficult to collect similar data.

## Methods

### Study design and participants

This study was a retrospective cohort study performed at the Osaka University Hospital, a tertiary academic medical facility in Japan. Data from routine medical workups for HCWs aged 18 to 65 years old were collected between 2001 and 2012. Of these, newly employed HCWs who had never been vaccinated against HBV and were confirmed to be negative for anti-HBs were included in this study. The need to obtain informed consent was waived because we retrospectively collected data without using any identifiable information of individuals and the application of any intervention.

### Laboratory testing and cut-off levels

Post-vaccination serologic testing for anti-HBs titer was performed approximately 4 to 5 months after the final dose of the primary vaccination or booster. The anti-HBs immunoassays were performed using the ARCHITECT^®^ system (Abbott Laboratories, Japan). The reliable lower limit of anti-HBs in this assay was taken as ≥10 mIU/mL, with an upper limit of 1,000 mIU/mL. Samples with anti-HBs levels over the upper limit were regarded as 1,000 mIU/mL. The cut-off level for the anti-HBs test was 16 mIU/mL, with titer of <16 mIU/mL being considered negative and ≥16 mIU/mL as positive. A booster dose of hepatitis vaccine was administered to individuals who tested negative for anti-HBs.

### Definitions

Primary vaccine response failure in this study was defined as a failure in producing protective levels of anti-HBs after completion of the primary 3-doses hepatitis B vaccination. Secondary vaccine response failure was defined as a decline in the level of anti-HBs below the cut-off after being elevated in response to the primary vaccination. An individual who obtained the protective titer of anti-HBs after primary hepatitis B vaccination was called a primary responder. A non-responder refers to a person who did not develop anti-HBs after completing the primary vaccination. Among the responders, we defined a long responder as one who retained protective levels of anti-HBs for 3 years or more after the primary vaccination. In contrast, persons whose anti-HBs titers showed negative conversion within 3 years after the primary vaccination were regarded as short responders.

### Vaccination schedule

Anti-HBs negative HCWs were subjected to primary HB vaccine, and the anti-HBs titers of primary responders were checked annually. Primary HB vaccine was administered with three doses of 10 μg recombinant HB vaccine Bimmugen^®^ (Chemo-Sero-Therapeutic Research Institute, Kumamoto, Japan) on time-point 0, 1, and 6-month. When anti-HBs levels declined to negative ranges, we administered a booster dose. The booster doses for those with secondary vaccine response failure were administered with either a single dose of Bimmugen^®^ or three doses of 10 μg of the heterovaccine Heptavax-B^®^ (Merck Sharp & Dohme, West Point, PA, USA). Data on the reasons for the recommended number of booster doses are not available.

### Statistical analysis

Statistical analysis was performed using EZR software, which is a modified version of R Commander (version 2.2–5) based on R (version 3.3.1). Kruskal-Wallis rank sum test and Mann-Whitney U test with Bonferroni *p* value adjustment were performed to compare initial anti-HBs titers after primary vaccination and the duration of its positivity. Additionally, a log-rank test for trend was performed to assess the relationship between the initial anti-HBs titers and the duration of positivity. To compare the rates of anti-HBs positivity at ≥5 years between single and 3-doses boosters administered to short responders, Fisher's exact test was applied. In all instances, a *p* value <0.05 was considered statistically significant.

## Results

**Fig 1:**
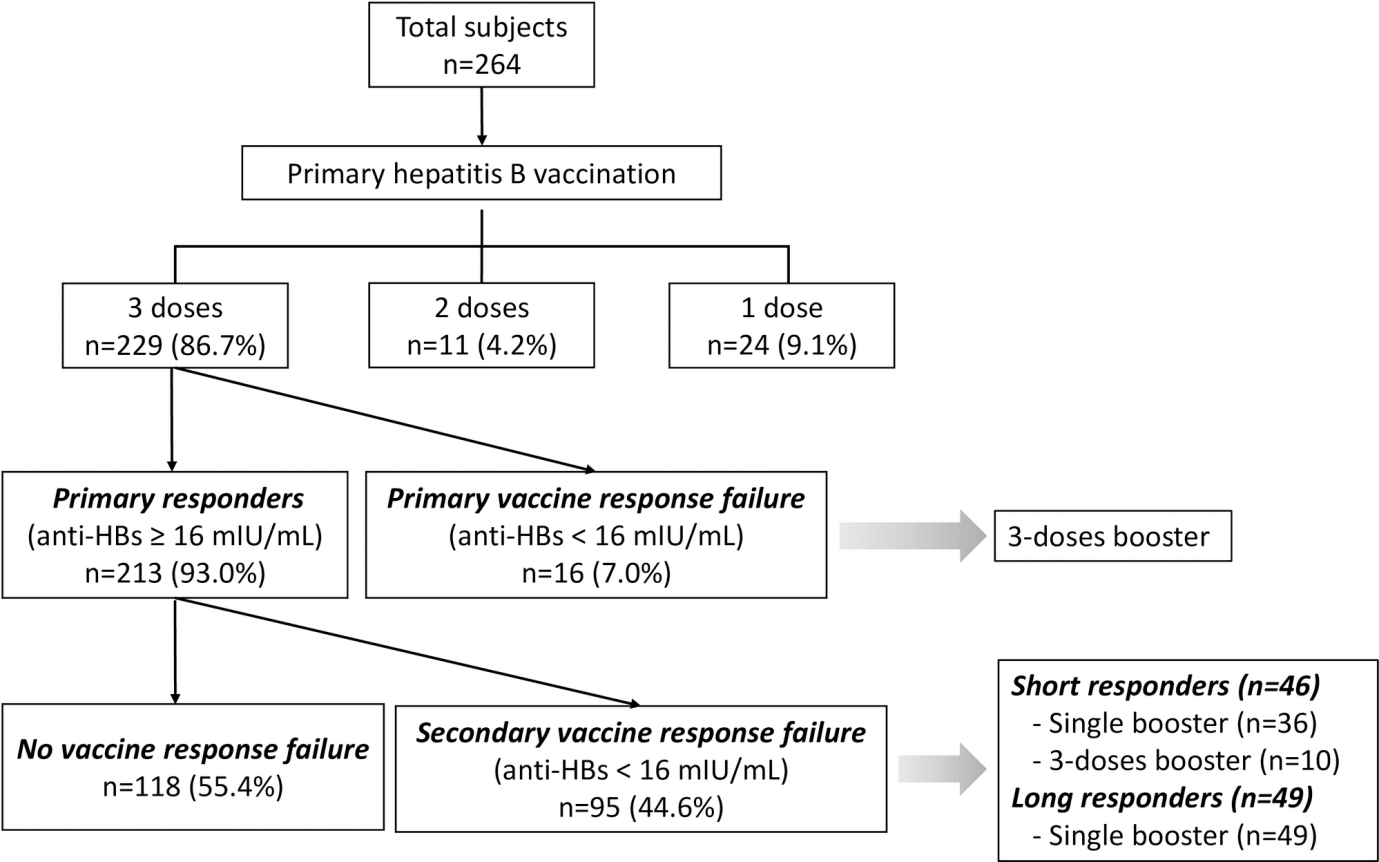
Flow of the study. Individuals who retained positive anti-HBs titers for >3 years were regarded as long responders and those who did not were regarded as short responders.

During the study period, data on 264 subjects were collected. Of these, 66 (25%) were men. The median age of the subjects was 25.3 years (interquartile range [IQR]: 23.1–31.7) years old, ranging from 20–58 years old. Although the completion of the full vaccination schedule was strongly recommended, 35 (13.3%) of the HCWs did not complete the schedule. Among those who received all three doses of the primary vaccination, 213 were primary responders (93.0%) and 16 were non-responders (7.0%). Among the primary responders, 95 cases of secondary vaccine response failure (44.6%) with 46 short responders and 49 long responders were observed. Among the short responders, 36 received a single dose, while 10 were given 3 booster doses. All the long responders received a single booster dose. The overall flow of the study is depicted in **Fig 1**.

**Fig 2:**
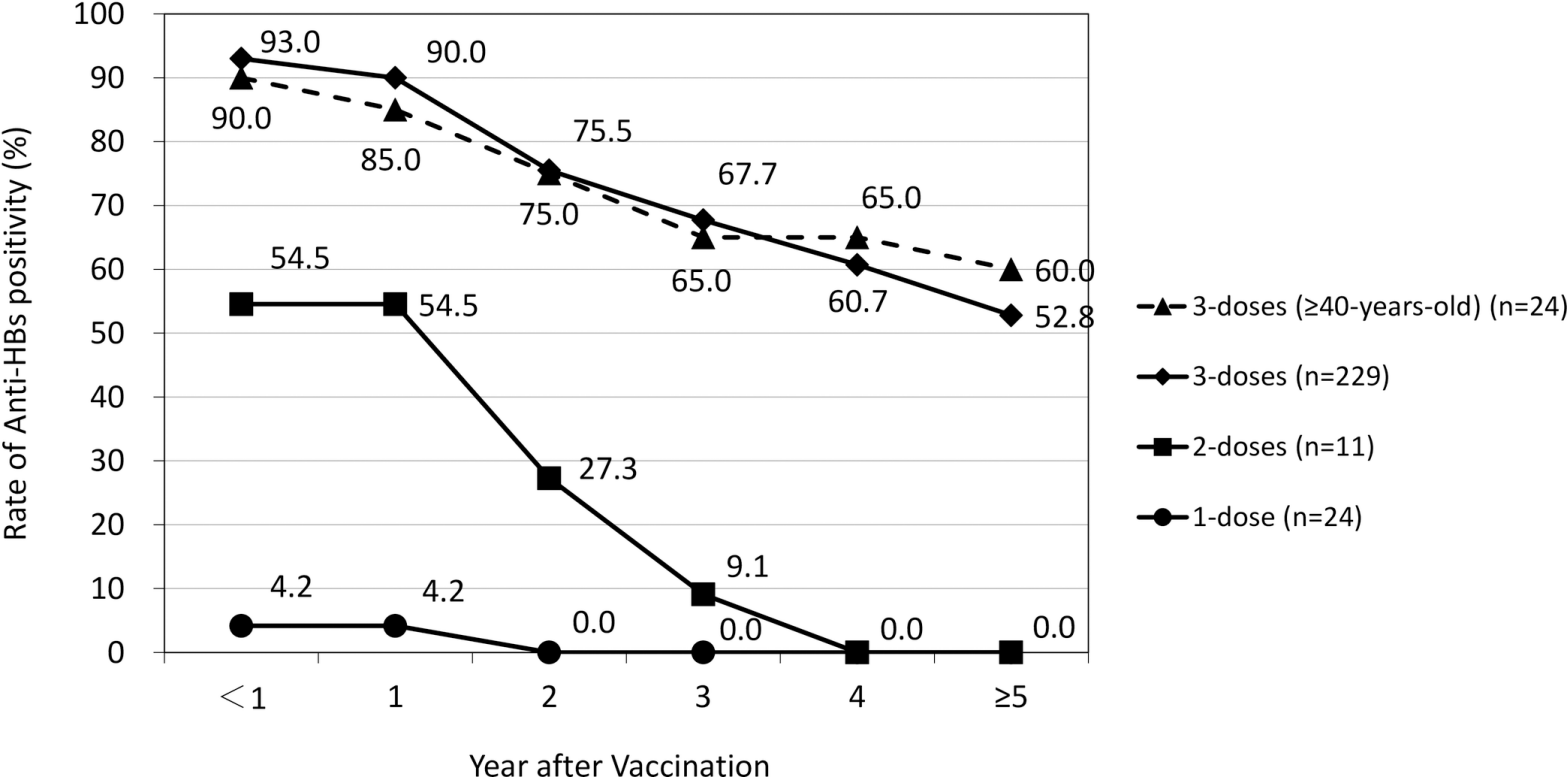
Doses of primary hepatitis B vaccine and rate of anti-HBs positivity.

First, the association between the number of primary HB vaccine doses and the rate of positivity and durability of anti-HBs was examined. The rate of anti-HBs positivity after the primary vaccination were 93.0% (n = 229), 54.5% (n = 11), and 4.2% (n = 24) after three, two and single doses, respectively. The anti-HBs titers chronologically decreased in each cohort, especially those induced by incomplete vaccination remained in the positive range for not more than 4 years. In contrast, more than half of those who received a complete vaccine series retained positive titer levels for over 5 years. This was also true for the population cohort aged > 40 years; the anti-HBs positivity was 90.0% after primary vaccination and declined similar to those in the overall population (**Fig 2**).

**Fig 3:**
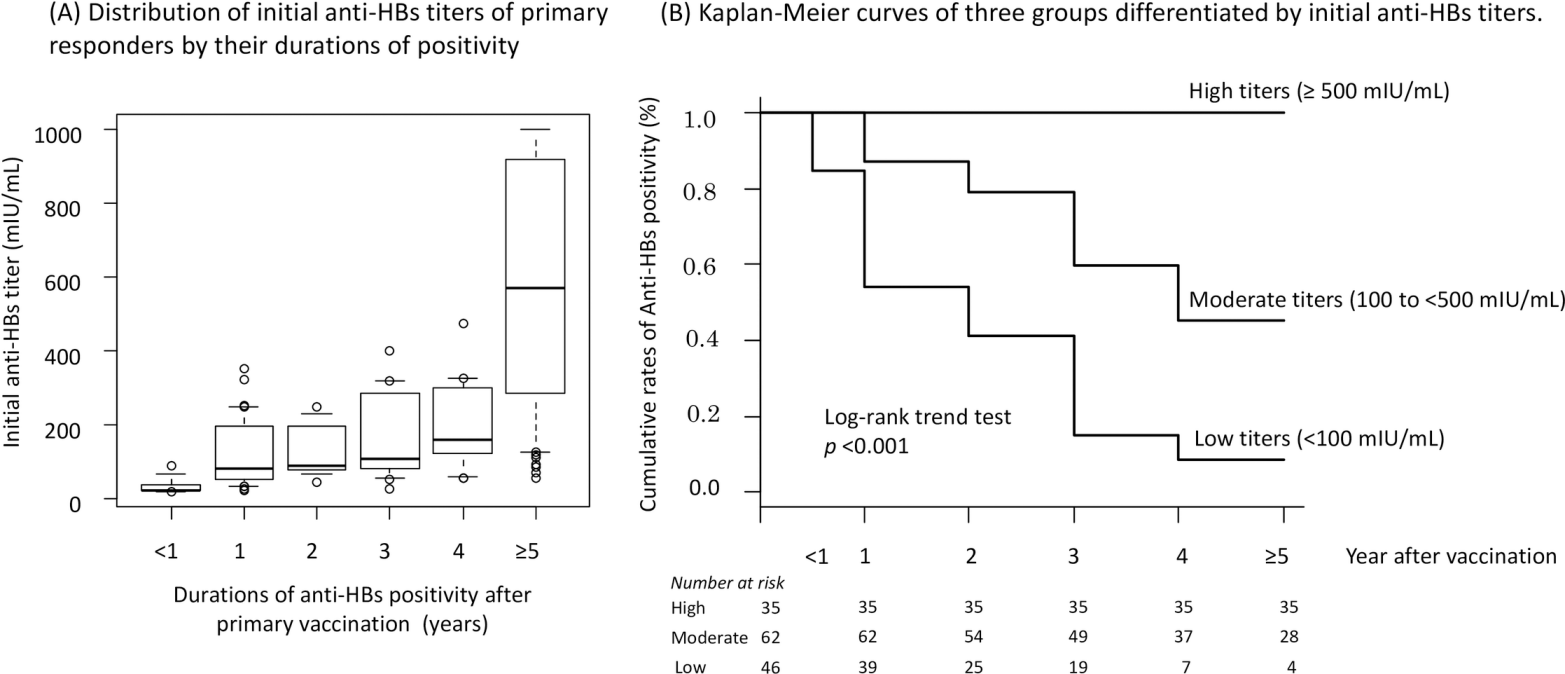
Initial anti-HBs titers of primary responders after full series of vaccination and its duration of positivity. (A) A box indicates a median and interquartile range, and error bars denote the 10th and 90th percentiles. (B) High (> 500 mIU/mL, n = 35), moderate (100–500 mIU/mL, n = 62), and low (< 100 mIU/mL, n = 46) titers. It was a significant trend that individuals with lower titers of initial anti-HBs experienced secondary vaccine response failure earlier.

Of the 213 primary responders, 143 had their serological tests performed according to the schedule, while 70 were excluded from the subsequent analyses since their serological tests were not performed as scheduled. The initial anti-HBs titers were categorized into the following groups based on their duration of positivity: <1, 1, 2, 3, 4, and ≥5 years, respectively. The number of individuals in each category was 7 (<1 year), 33 (1 year), 11 (2 years), 13 (3 years), 12 (4 years), and 67 (≥5 years). The median (IQR) titers of initial anti-HBs in each category were 22.2 (20.1–36.2), 81.6 (51.1–195.0), 89.1 (75.0–195.1), 106.7 (81.4–285.1), 159.2 (130.0–293.0), and 569.6 (234.5–919.2) mIU/mL, respectively. Individuals with longer anti-HBs positivity durations tended to have higher initial anti-HBs titers. The result of the Kruskal-Wallis rank sum test was statistically significant between these categories (*p*<0.001). The *p* values obtained when comparing the <1 year group with others were as follows: 0.033 for 1 year, 0.028 for 2 years, 0.017 for 3 years, 0.013 for 4 years, and <0.001 for ≥5 years. Additionally, *p* values obtained when comparing the ≥5 year group with the others were <0.001 in each case (**Fig 3A**).

Kaplan-Meier curves were drawn by categorizing the primary responders into three groups according to their initial anti-HBs titers: high (> 500 mIU/mL), moderate (100–500 mIU/mL), and low (< 100 mIU/mL) (**Fig 3B**). The log-rank trend test revealed that a statistically significant correlation between individuals with lower titers of initial anti-HBs and the earlier occurrence of secondary vaccine response failure. None of the individuals acquiring anti-HBs titers >500 mIU/mL after primary vaccination recorded levels below the protective range for at least 5 years post-vaccination.

**Fig 4:**
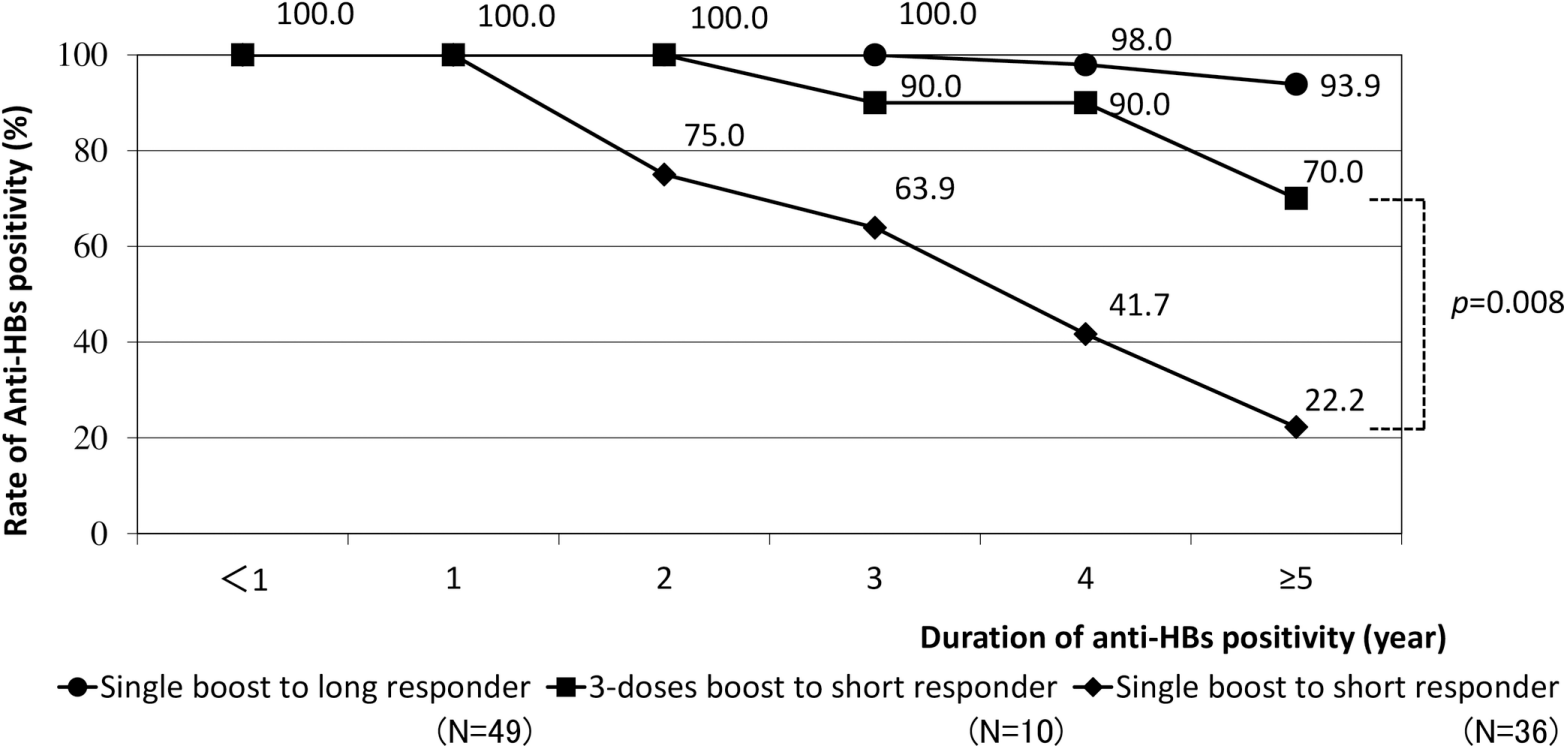
Booster doses administered to short/long responders and duration of anti-HBs positivity.

Additionally, booster doses required for both short and long responders were investigated. As shown in **Fig 4**, majority (93.9%) of the long responders maintained positive titers after 5 years only with a single additional dose. In contrast, 70.0% of short responders with a repeated complete vaccination series retained positive anti-HBs titers after 5 years. Of note, the rate of anti-HBs positivity after administering a single dose of booster vaccine to short responders decreased progressively over time. There was a significant difference in the rates of anti-HBs positivity between administering single boosters and 3-dose boosters to short responders at ≥5 years (*p* = 0.008).

## Discussion

The main finding of this study is that short responders need another booster series (3 doses of HB vaccine) to maintain the protective levels of anti-HBs, while long responders, who maintained positive anti-HBs titers for more than 3 years, could regain long-term anti-HBs positivity with a single booster dose. Chronic HB can result in *de novo* hepatitis that may be extremely fatal [18]. Thus, it would be preferred that HCWs maintain higher serum anti-HBs levels than previously considered to prevent occupational infections. However, the durability and robustness of the immunity provided by HB vaccine in adults and an optimal strategy for administering additional doses to individuals with secondary vaccine response failure have not been well stipulated. Our results may be helpful in considering a safer and more efficient HB vaccine strategy for HCWs in future.

Post-vaccination anti-HBs levels decline over time. However, there are limited data on the durability of protective anti-HBs levels in HCWs who were not immunized during childhood. Previous studies have reported adequate anti-HBs levels in 23% to 48% of adults, within 3–18 years post-vaccination [19–24]. In a recent study that examined the durability of anti-HBs in vaccinated adults and their response to a booster, anti-HBs levels were found to decrease below the protective level after 10–31 years in 25% of the subjects [24]. In our study, 52.8% of vaccinated HCWs retained positive anti-HBs levels for 5 years after the primary vaccination. A long-term prospective cohort study is warranted to obtain data that are more accurate.

The need to administer vaccine boosters to those with anti-HBs sero-negative conversion remains controversial. Although the authorities deny its necessity [9, 10], this would pertain to developed countries like the United States and Europe, where there is a relatively low prevalence of HBV infection and well-established infection control activities. However, it is questionable whether a similar approach is reasonable in other regions with high prevalence of HBV. In high-risk countries like Asia, Africa, and South America, it may be safer to implement an elaborate HB vaccine schedule for HCWs, where anti-HBs titers of vaccine responders are measured annually or periodically and booster vaccines are administered according to the duration of their primary anti-HBs elevation. Even in the developed regions, HCWs with frequent exposure, such as those in the surgical or dialysis departments, may also benefit from the deliberate vaccine strategy.

Our study provided other interesting perspectives to be discussed. First, higher initial anti-HBs titers may promise a subsequently prolonged duration of protection in HCWs. In children aged 6 months or older, initial anti-HBs titers after primary vaccination were associated with the persistence of higher anti-HBs levels at 15 years [11]. Similarly, our statistical analysis demonstrated a significant relationship between initial anti-HBs titer levels and the duration of positivity. Second, completion of the primary 3-dose vaccination was essential for achieving a higher titer of anti-HBs and longer duration of positivity. The rate of anti-HBs positivity among those who completed the primary vaccination schedule was 93.0% in our cohort, as previously reported [25]. Subsequent declines in anti-HBs titers were conspicuous among those who had not completed the vaccination schedule, as shown in **Fig 2**. Thirdly, we found that the HB vaccine was as effective in the elderly as the general population In previous reports, the immunogenicity of the recombinant HB vaccine was seen in 85.3%, 56%, and 45.7% of individuals aged >40 years [26], >50 years [27], and >60 years [28], respectively. Due to the small sample size, further studies are needed to reach a conclusion.

This study has some limitations. First of all, baseline testing for HBV markers such as anti-HBc, HBsAg or HBV-DNA were not performed. Thus, some individuals with chronic HB, in whom anti-HBs poorly reacts to the vaccination, could have been included in the study and compelled to undergo unnecessary repeated testing and booster vaccinations. Second, the backgrounds of HCWs were not considered. Individuals with chronic diseases, immune defects, on immunomodulatory medications, or even smokers, alcoholics, obese persons, and males have a diminished immune response following vaccination [9, 29, 30]. The influences of these possible confounding factors were not adjusted for in the present study. Thirdly, post-vaccination serologic testing was performed only four or five months after the administration of the final dose, despite the recommendation that testing should be performed within 1–2 months post-vaccination. Finally, due to the non-random nature of the study with a relatively small sample size, the result should be regarded as preliminary data that needs further investigation.

In conclusion, it may be useful to differentiate HB vaccine responders based on their primary anti-HBs positive durations and boost them accordingly to maintain anti-HBs titers efficiently. The elaborate vaccine program may be beneficial to high-risk populations, especially in regions with high HBV prevalence. The small size and nonrandom approach of the study, however, limit the interpretation of the results. There is the need to perform a randomized, large-scale, multi-center study that entails HBV baseline data.

## Supporting information

S1 Fig Anti-HBs titers after booster of hepatitis B vaccination. Anti-HBs titers were examined in those with sero-negative conversion after a primary full series of HB vaccine. Their anti-HBs titers on day 0 were 2.0, 4.5, 6.6, 9.0, and 9.0 mIU/ml in order. Post-revaccination, the titers were frequently checked to observe the time to rise of anti-HBs. As a result, elevations of anti-HBs over the protective level were observed in 4 cases by the 7th day, and 1 case by the 8th day. Vaccination and measurement of anti-HBs were performed as noted in the Method section of manuscript. Tests for HB surface antigen and antibodies to HB core antigen yielded negative results among the HCWs.
(TIF)
