# Supplementary figures and images for: Durability of immunity by hepatitis B vaccine in Japanese health care workers depends on primary response titers and durations
Source: PLoS One. 2017 Nov 9;12(11):e0187661. doi: 10.1371/journal.pone.0187661 (PMC5679562; doi:10.1371/journal.pone.0187661)

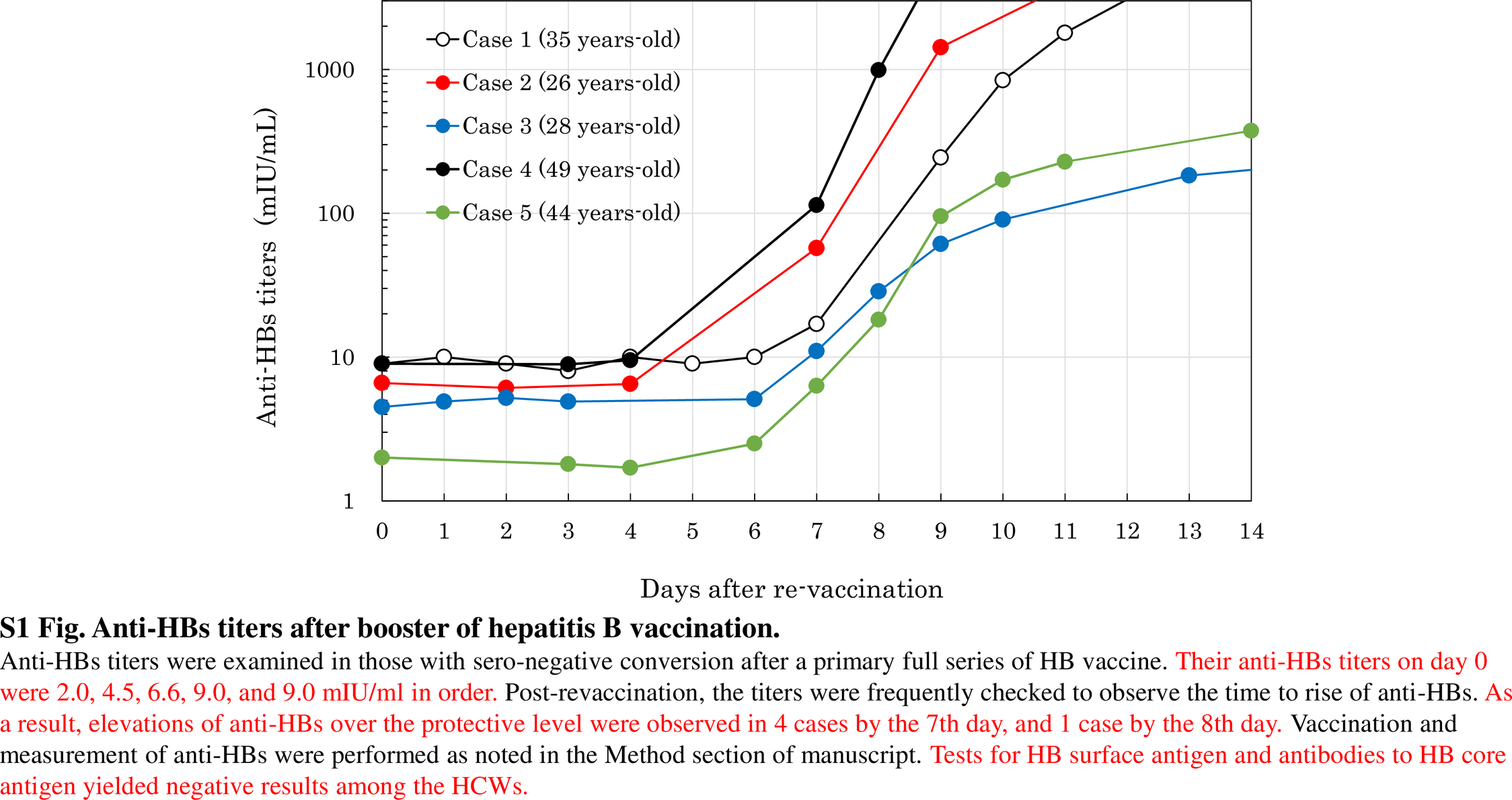

Supplement: S1 Fig — Anti-HBs titers were examined in those with sero-negative conversion after a primary full series of HB vaccine. Their anti-HBs titers on day 0 were 2.0, 4.5, 6.6, 9.0, and 9.0 mIU/ml in order. Post-revaccination, the titers were frequently checked to observe the time to rise of anti-HBs. As a result, elevations of anti-HBs over the protective level were observed in 4 cases by the 7th day, and 1 case by the 8th day. Vaccination and measurement of anti-HBs were performed as noted in the Method section of manuscript. Tests for HB surface antigen and antibodies to HB core antigen yielded negative results among the HCWs. (TIF) [file pone.0187661.s001.tif]
